# A Family Poisoned by Eating a Garr

**Published:** 1851-03

**Authors:** W. Brooks

**Affiliations:** Circleville, Illinois


### ARTICLE IX.

*A Family poisoned by eating a Gar.* By Dr. W. Brooks, of Circleville, Illinois.

Amid the many and varied forms of diseased action the destructive ravages of which, the Physician is called upon to aid in counteracting, it is rare to meet with such a case as I am about to relate.

April 27, 1850, I was called at 1½ o'clock A. M., to attend upon a family residing in this village, said by the messenger to have been poisoned. I immediately hastened to their assistance, and on entering their apartments the scene that presented was one well calculated to elicit the sympathies of the most obdurate heart. There were no less than five persons suffering in all the agony that pain could inflict. I inquired into the cause and got the following history: A party had been on a fishing excursion the day before, and had been so unsuccessful as to return with nothing but what is vulgarly called a Gar, (a species of fish quite abundant in many of the western rivers, which has a long pointed horny mouth like the shark, and a hard scaly skin.) The family being anxious for fresh fish, the Gar was skinned, dressed and par-boiled, it together with *the eggs* were then fried and served up for the table. The whole family partook heartily of the rare delicacy and admired the dish. At 11 o'clock in the evening they felt a burning sensation in the pit of the stomach, which was soon succeeded by vomiting, purging, and cramping, in their most aggravated forms. The pulse was small and frequent. Tongue somewhat swollen, with a red serrated margin, and a dark yellow fur along its centre. The matter ejected from the stomach was very green, and very offensive. Stools not dissimilar in appearance from those of a case of dysentery. Cold sweats, alternating with flushed and suffused countenance.

*Treatment.* I ordered a sinapism applied to the epigastrium, and administered the following: ℞ camph. 3 grs., Flos. sulph. 3 grs. opii ½ gr. The first second and third doses were rejected as soon as they came in contact with the irritated stomach, the fourth was retained longer. I found that they were effecting some good, and persisted in their use, giving another as soon as the previous one had been ejected from the stomach. Within an hour from the time I gave the first dose of the above mixture, all of the alarming symptoms had, excepting one case, disappeared. I left orders for the powders to be given every two hours until I should see them again.

At 10 o'clock the same day, all better but the one that seemed most obstinate to manage in the morning. This Patient vomits and purges still, occasionally, with some fever, fured tongue, and offensive breath, complains of pain in the stomach and bowels. Prescribed cal. camph., and sulph. morphia in small doses every two hours.

28th. Found patient this morning in a comatose state, but had rested easy all night. Pulse hardly perceptible at the wrist, free from all pain, the surface of the body, hands and feet, very dry. Tongue slightly furred and a foul breath.

Prescribed cal. camp. and morphia, in order to procure an action from bowels, as there had been no evacuation from 4 o'clock on the previous evening. This to be followed by enemata should it be deemed necessary.

29th. Patient this morning suffering only from general prostration, put her on Dover's powders and quinine, and dismissed her as cured. I had neglected to say that my other patients we're similar in most respects in symptoms and treatment to the one already described. The foregoing, peculiar and novel as it is, nevertheless is a true "fish story."

